# National-level key stakeholders’ perspectives regarding intervention progression and emerging challenges within the national stillbirth reduction response in Uganda

**DOI:** 10.1371/journal.pone.0285172

**Published:** 2023-04-28

**Authors:** Eric Ssegujja, Michelle Andipatin

**Affiliations:** 1 Department of Health Policy Planning and Management, School of Public Health, College of Health Sciences, Makerere University, Kampala, Uganda; 2 School of Public Health, Faculty of Community and Health Sciences, University of the Western Cape, Cape Town, South Africa; 3 Department of Psychology, University of the Western Cape, Cape Town, South Africa; University of Health and Allied Sciences, GHANA

## Abstract

**Introduction:**

Implementation of evidence-based interventions was adopted to respond to the stillbirth burden from the global campaigns. However, new challenges emerge in the process of rolling out such interventions into routine services more so in the context of resource-limited settings. Since the scale-up of policy recommendations to address stillbirth in Uganda, the health system response has seldom been explored. This study was conducted among national-level key stakeholders to elicit their perspectives regarding intervention progression and challenges emerging from the implementation of the national stillbirth reduction strategies in Uganda.

**Methods:**

The study adopted an exploratory qualitative design with interviews conducted among a purposively selected sample of national-level actors drawn from the maternal and Child Health (MCH) policy networks. Respondents were primed with ongoing national-level stillbirth reduction strategies as a case and later asked for their opinions regarding intervention progression and emerging challenges. All interviews were conducted in English and transcribed verbatim. Atlas. ti was used to facilitate the coding processes which used a pre-determined codebook developed a priori based on the applied framework. A thematic analysis technique was used.

**Results:**

Human resources as reflected in the slow recruitment of essential staff, motivation and attitudes of the available human resource, on and off-drug stockouts, and equipment interruptions posed challenges to the effective implementation of interventions to address the stillbirth burden. The policy translation process was sometimes faced with deviations from the recommended practice. Deviations from guideline implementation, inadequate managerial skills of the health workers and managers in stewarding the implementation processes, inadequate implementation feedback, loops in communication and working with a passive community also posed process-dependent bottlenecks. Outcome expectation challenges stemmed from the inability to deliver stillbirth reduction interventions along the Reproductive Maternal New born Child and Adolescent Health (RMNCAH) continuum of care and the overconcentration of facility-level intervention with less focus on community/demand side interventions.

**Conclusion:**

In this exploratory study, national-level stakeholders perceive the adopted stillbirth reduction strategies as having the potential to address the burden. They, however, highlight potential challenges along the input-process-outcome continuum which ought to be addressed and opportunities to explore potential solutions befitting the national-level context.

## Introduction

Globally, over two million pregnancies end in stillbirth every year [1]. This burden is disproportionally higher in low- and middle-income countries (LMIC) in Africa and Asia [2]. Although stillbirth risks are known to manifest along the life course of adolescent girls and women, pregnancy and childbirth present the heightened period when most stillbirth risks occur [3]. It is a devastating period for the parents, family, health workers and the entire health system that is expected to avert such negative outcomes [4]. Efforts towards addressing this public health challenge have seen different strategies translated into national policies for better Reproductive Maternal New born and Child Health (RMNCH) service delivery [5]. While a couple of interventions targeted at stillbirth reduction have been introduced into the health systems, challenges are encountered during their everyday implementation processes.

Stillbirth reduction strategies include those that specifically target stillbirth risk factors [5] and others that target the general improvements in the health systems specifically as it relates to the RMNCH service delivery [6]. There are some interventions geared towards addressing the social determinants of health such as the demand side factors that impede the optimal utilisation of available RMNCH services [4, 7]. Their implementation is supported through policy and guidelines that spell out the processes through which such interventions are translated into service delivery. However, ensuring their proper implementation for optimal delivery of quality RMNCH services remains an elusive target [1, 3, 6]. This is especially true when implementation occurs in resource-limited settings like Uganda.

Within the Ugandan context, despite improvements in the utilisation of the first Antenatal Care (ANC), the numbers are reported to decline when it comes to the completion of the recommended 4 ANC visits [8–10]. The numbers continue to drop further for those that deliver within the health facilities by skilled birth attendants when compared to their overall antenatal care attendance [11, 12]. The same is observed when it comes to notification of perinatal deaths [13, 14]. Although it is now a policy requirement for all deaths occurring within the facilities, the country is yet to realise full implementation where all perinatal deaths are notified. Even for the few that get notified, completion of their review and entry of results into the District Health Information System (DHIS) events report remains a challenge [15]. This frustrates the national efforts towards the reduction of the stillbirth burden through responding to the likely causes.

Ensuring universal coverage for policies responding to stillbirth in Uganda remains a challenge. Implementation level bottlenecks are some of the reasons to explain the slow attainment of universal coverage for policy implementation [16]. Delivering quality RMNCH services is an overarching goal for the health systems in Uganda [15]. Different approaches include the establishment of the Quality Assurance Division at Ministry of Health (MoH), Continuous Quality Improvement guidelines, the National Quality Improvement Conference for experience sharing, the Health Facility Quality Assessment Program (HFQAP) and 5S-Continuous Quality Improvement (CQI) at Regional Referral Hospitals (RRH) and in districts to ensure and monitor quality service delivery exist. Several models and frameworks exist to guide processes of ensuring quality in health service delivery [17]. The Donabedian model is one such framework that guides assessments of quality in health services [18]. It approaches quality from the inputs, processes and outcomes expectations of the services delivered. It considers multiple lenses when conducting these assessments including the performance of the health workers, the contribution of the service users and the response from the health systems [19].

Global campaigns to address stillbirth were well received and translated in Uganda and our earlier results showed that they received good political prioritisation [20] which facilitated timely translation into national policies and guidelines. Different aspects of the global campaign recommendations were adopted in line with long-standing health systems strengthening aspirations covering the entire spectrum of the health system building blocks. Embedding into policies and popularisation of high-impact evidence-based intervention among health workers was at the forefront. Practices such as neonatal resuscitation, perinatal death reviews, use of partograph, operationalisation of Comprehensive Emergence Obstetric Neonatal Intensive Care (CEmONIC) and above all promotion of uptake and early initiation of ANC involved improving health facilities into mother-friendly centres for positive ANC experience. It would later be accompanied by the popularisation of the 8 ANC contacts with community structures playing a crucial role in the identification, referral and follow-up. Other interventions were around improvements in data systems where stillbirth was included within the national surveillance system as a notifiable condition which mandated the health workers to report incidence within 24 hours, rolling out the birth and death registration to estimate burden and having stillbirth as a performance and quality indicator at district and health facility respectively. There were interventions targeted at improving health worker skills such as improving the Doctor-patient ratio which saw their recruitment at Health Centre (HC) IV to specifically target Maternal and Child health (MCH) service improvements, in-service training for workers for skills improvement such as resuscitation, training and recruitment into service of rare cadres such as anaesthetists for CEmONIC and special recruitment of midwives to match service delivery demands. Other interventions targeted infrastructure such as the expansion and construction of new maternity wards, operating theatres, neonatal intensive care units, and upgrade of health facilities to match the MCH service delivery mandate among others.

Subnational-level health systems responded by adapting recommendations into their routine service delivery [21] with health workers too leveraging resources within their midst including support from professional networks to ensure proper implementation [22]. Despite variations in implementation along the way from the national to the subnational level [23]. However, challenges continue to be encountered with anecdotal evidence documenting some of the shocks that make it difficult for the health systems to respond to all stillbirth risks [15]. Challenges to the delivery of health services have elsewhere been documented in the literature [8]. These range from inadequate human resources, wastage of available human resources due to absenteeism, rampant drug stockouts, and inadequate funding among others [24]. However, these are mainly reported concerning general MCH service delivery. Interventions addressing stillbirth likely face unique challenges and we aimed to explore this in detail. The aim of this paper was therefore to gain a deeper understanding of the intervention progression and the challenges experienced and to foster an understanding of prevailing experiences.

Having introduced and implemented high impact evidence-based interventions into the health systems to address stillbirths [15]. This study, therefore, examined the national-level key stakeholders’ perspectives on intervention progression and emerging challenges towards the national stillbirth reduction strategic response in Uganda. Data collection was conducted before the Covid-19 lockdown in the country, therefore, its effect on the health systems although acknowledged in many subsequent writings did not in any way influence the participants’ responses.

## Materials and methods

### Study design

Details about the applied methodology have been described elsewhere [20] but briefly; The study adopted an exploratory qualitative design conducted in Uganda among national-level key informants drawn from the existing maternal and child health policy networks. This manuscript aimed to capture national-level perspectives regarding the implementation processes of policies and interventions to address the national stillbirth burden. It took a qualitative descriptive approach to gather a deeper understanding of how these interventions were unfolding and the emerging challenges that were being confronted from the national-level stakeholders’ perspectives.

### Study setting

**Fig 1:**
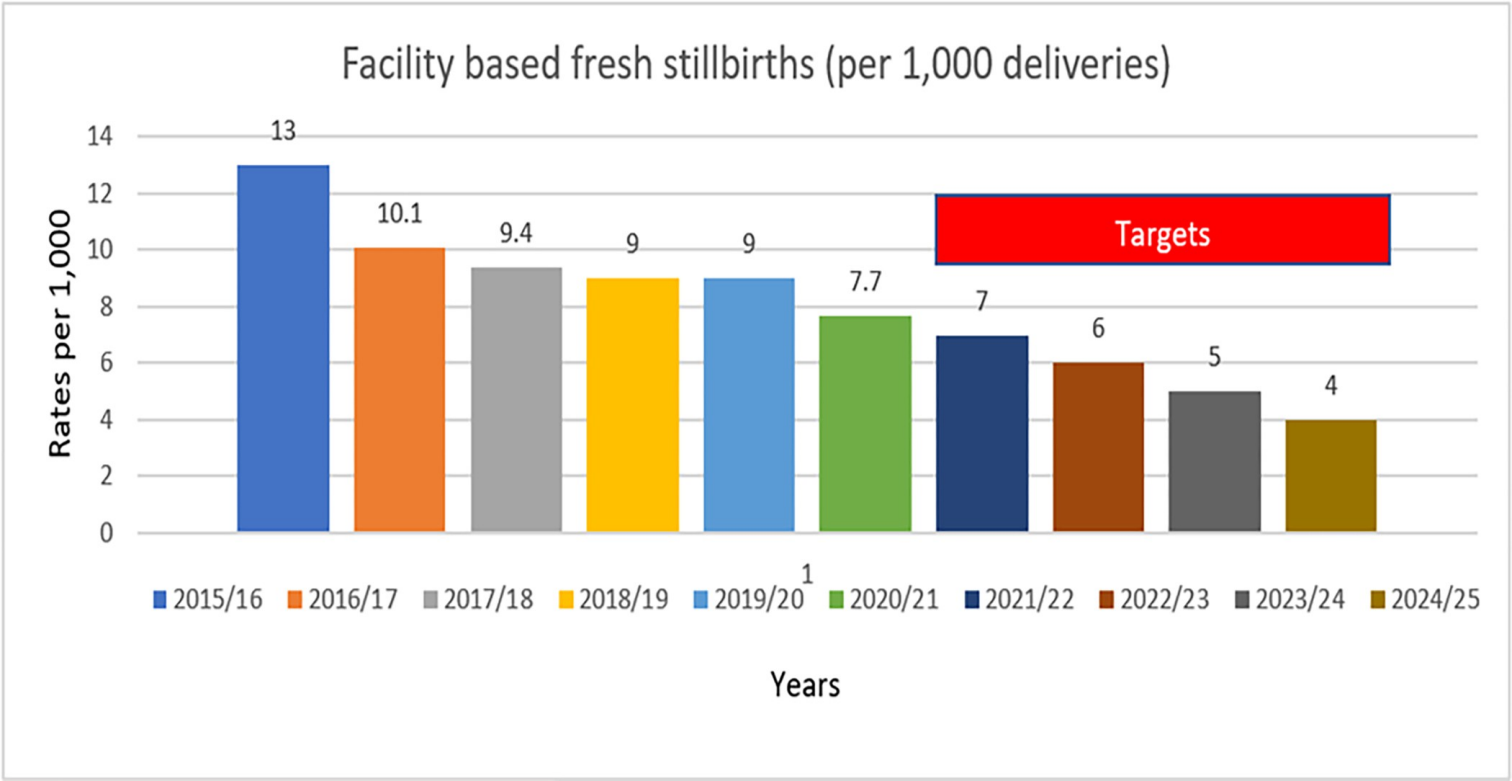
National stillbirth rates and targets over the years. Source: Ministry of Health, Uganda.

The study was conducted in Uganda at the national level. The country enjoys substantial external support through development aid with the health sector as one of the leading beneficiaries. Most recently, donor support for health accounted for up to 43% of the total health budget with much of it off-budget support [15]. RMNCH is one of the key programs’ beneficiaries of this donor support. The country adopted drastic measures as reflected in policy alternatives to address the national stillbirth burden as advocated for during the global campaigns from the early 2010s and have been integrated into national RMNCH policies [15]. Conceptually these policy recommendations originated from; 1) World Health Organisation (WHO) recommendations, 2) local evidence generated from donor-supported projects, and 3) insights from routinely collected data and implementation experiences by the Ministry of Health (MoH). The overarching policy expectation is to promote deliveries and antenatal and postnatal care within health facilities. These have been reflected as strategic interventions for health under National Development Plan (NDP) III [15]. Broadly they include; improving maternal neonatal, child and adolescent health at all levels of service provision, improving the functionality of the health system to deliver quality and affordable health care, and improving social determinants of health, Universal Health Coverage (UHC) as some of the key result areas for monitoring. The country has registered substantial progress in reduction of facility-based fresh stillbirths currently standing at 7.7/1000 and is on course to achieve the national target of 4/1000 by 2025 as reflected in Fig 1. There are regional variations in Institutional Perinatal Mortality Rate (IPMR) with Kampala disproportionately performing poorly at 40.2/1000 while Teso region has the lowest at 14.5% compared to the national target of at least 15/1000 [15]. Overall, there have been progressive improvements in performance regarding perinatal death notification and reviews over the past five years (2016–2021). Of the total perinatal deaths reported, up to half (50.8%) were notified within the recommended 24 hours and only 41.2% were reviewed and entered into the DHIS event report [15]. Regional variations exist where the Kampala region registered the highest notification rates at 100% while Tooro only reported 14.2% of the total cases within the recommended time. These variations are also visible when it comes to perinatal death reviews where Ankole region had the highest review rates at 75.9% while Bunyoro had the lowest at 8.8% of the national target of at least 14% [15]. It is within this context that this study was conducted.

### Study population and sample

The study population comprised purposively selected respondents drawn from the national-level MCH policy networks. Potential respondents were pre-identified as eligible with guidance from individuals familiar with national-level MCH programming and policy processes. A list was generated with the contacts from which they were approached. Additional respondents were selected based on leads from the Ministry of Health, fellow respondents and information from reports detailing their contribution to national-level policy processes. Overall 20 participants were interviewed. We employed a process tracing methodology together with purposive sampling to identify key documents for review.

### Data collection procedures

Data collection was conducted between March and June 2019. It primarily consisted of two methods which included key informant interviews and document reviews. The exercise was conducted by the first author (ES) with support from two graduate-level research assistants who were experienced in qualitative methodologies. At the design stage, the inclusion criteria were set to include only those respondents who were familiar with national-level MCH policy processes and had at least spent two years in their current positions. An initial list of potential respondents was pre-generated from where the final sample would be drawn. They were approached through telephone calls where the aims and objectives of the study were explained. For those that agreed to participate, a day and time of the interview would be agreed upon. Two callbacks were made in case the respondent was not available during the first attempt after which a replacement would be identified. Face-to-face interviews lasting from 45 minutes to one hour depending on the point of saturation were conducted at the respondents’ places of work. During the interviews, they would be asked to recommend any other potential respondent whom we could also include in our sample. These were snowballed and contacted for possible participation after the commencement of the study. An interview guide consisting of open-ended questions developed for this study was used. On the day of the interview, a safe and secure place would be identified from where the interview would be conducted. Field notes were taken at the end of each field day during the daily debrief meeting attended by the data collection team. Overall 20 out of the 23 respondents who had earlier on been contacted took part in this study the point at which we attained saturation. The three who had earlier expressed willingness to participate could not be accessed after the third callback. 17/20 were female and drawn from Professional associations (6), the Ministry of Health (5), Non-Governmental Organisations (NGOs) (4), Private not for Profit (2), Academia/Researchers (2) and the Private-for-Profit sector (1).

The document review exercise was conducted by the first author with key documents reviewed including Ministry of Health reports and Strategic Plans, and project reports from donor-funded projects. A grey literature search was conducted on the internet and it prioritised websites of leading organisations, projects and donors involved in national-level programs to address the stillbirth burden.

### Analytical framework

Translation of policy and delivery of interventions aimed at responding to the national level stillbirth burden was conceptualised from the perspective of their implementation as intended. This would therefore be achieved through the provision of quality RMNCH services guided by evidence-based interventions as recommended by the relevant policy prescriptions. We, therefore, adopted the Donabedian model of quality healthcare service delivery as a guiding conceptual framework [18]. It approaches quality in terms of inputs, processes and outcome expectations. We analysed stakeholders’ views regarding the inputs that went into the implementation of these interventions in terms of the health systems building blocks such as trained health workers, financing and availability of medical products and supplies. The processes as viewed from technical quality by the service providers (guideline adherence) and perceived social quality from the service users’ perspectives for example user experiences during their interface with the health systems. The outcome expectations considered the perceived effectiveness of policy alternatives in addressing the stillbirth burden and effective coverage in terms of universal access for all as reflected in the overall effect of the stillbirth burden so far. The three aspects accounted for the overall health systems readiness to address implementation challenges to deliver interventions that would respond to the stillbirth burden in Uganda.

### Data management and analysis

All interviews were conducted in English and recorded verbatim using a digital audio recorder. They were then transcribed using Microsoft office word by two research assistants who took part in the data collection exercise and stored in a secure folder on a password-protected laptop computer only accessed by the study team. All transcripts were read through by the first author before exporting them to Atlas. ti a qualitative data management software for coding [25]. Using the guiding framework, each of the aspects of the Donabedian model was entered a priori as a code with its sub-codes to aid the coding process following a deductive approach. Text relating to a particular code was identified from the transcripts, highlighted, dragged and dropped in the respective codes in the process of assigning textual data to codes. Thereafter query reports were run for each of the codes using a query tool function within the software. a manual pile sorting exercise was done for each of the queries by reading through each of the texts to identify underlying meanings. Text with similar or related meanings was then grouped into separate piles. Each pile was then read through once more to identify underlying themes and sub-themes. A similar exercise was followed while analysing textual data from the documents that were reviewed. We then used the Consolidated Criteria for Reporting Qualitative Results (COREQ) checklist to guide the reporting of results with typical quotes used to bring out the respondents’ voices. Triangulation was done through a comparison of results from the different respondents’ categories and data collection methods.

#### Ethical approval and consent to participate

The study was performed following the Declaration of Helsinki. Ethical approval was granted by the Ugandan National Council for Sciences and Technology (SS 4575) while the study received a scientific review from the University of the Western Cape, Biomedical Research Ethics Committee (BM/17/9/1) in South Africa and, from the Makerere University School of Social Sciences Research and Ethics Committee (MAKSS REC 12.17.110) in Uganda. Individual written/verbal consent was obtained from all respondents before conducting the interview.

## Results

### Perceptions

#### Overall good reception of interventions in the health systems

Following the interventions that were introduced into the health systems to address inherent challenges, respondents noted these improvements were timely with the health systems ready to adapt them for better quality MCH services delivery. There was a general perception that the rolled-out interventions in the health systems to address the stillbirth burden had improved the practice and quality of MCH service delivery. By notification through the surveillance systems, MoH was able to learn at the earliest the stillbirth occurrence as information was shared from the sub-national level practitioners. Improvements in the data systems were reported to facilitate the establishment of the subnational and national level stillbirth burden and subsequent trends using timely data. Through the implementation of Maternal and Perinatal Death Surveillance and Response (MPDSR), health workers were able to establish the cause and support to work around the available interventions to respond to the identified gaps and risk factors. A recognition of the generalised service delivery improvement was observed. Despite this, it was also noted that some indicators persistently performed poorly compared to others. A case in point was reported about neonatal mortality.

### Inputs

#### Human resource-related challenges

The pace at which human resource training and recruitment into public service were not matching current population growth and subsequent health service demands which affected service delivery. It emerged that whereas efforts had been initiated to increase the numbers of trained health workers, the pace of recruitment into public service was noted to be slower than the rates of population growth which affected the implementation of interventions to address stillbirth risk among others;

*You see training is not the problem*, *the problem is the systems that are not responding to our population growth and because of that our current system*, *our population is outstripping the services that we currently have…… there are approximately 1*.*7 million pregnancies every year but our facilities are not designed to provide services for the 1*.*7 million*. ***#5 Professional association***

Consequently, it affected service delivery at the frontline due to increased workload among the few available staff and pressure on other resources which was likely to compromise the effectiveness of policy translation;

*human resource is a bigger challenge*, *I mean you are looking at one midwife who is attending to mothers in the labour ward and mothers in the ANC are waiting*, *some have come for immunization and of course*, *they would like family planning*. ***#3 MOH***

#### Cost of human resource

The cost of human resources was another challenge. It emerged that the inability to meet the staffing requirements or the attempts to improvise resulted in other unforeseen challenges. A case in point was the scarcity of midwives in some postings generally and the inability to recruit more into service. It emerged as a common thread among both the public and private sectors. Within the private sector, many of the private maternity service providers were unable to employ full-time midwives to offer services which had the potential to affect policy translation as reflected in the quote below;

*you see what happens is that when it comes to pregnancy and delivery*, *the best [at service provision] are the midwives but midwives in this country are few and then you have the many public demands and because they are few they are even expensive*. *Most of the Private-for-profit clinics can’t afford a full-time midwife and so the issues of human resources are mega and they need to be looked at*. ***#4 NGO***

Offering technical skills in absence of motivated health workers was another challenge in addressing stillbirth risk factors. The need to also pay attention to the intrinsic motivation of these health workers was raised as another area for attention. This would help address some of the other health system’s challenges. Other areas to boost intrinsic motivation for human resources for effective service delivery include; accommodation and a better working environment among others.

*Medical personnel are not motivated*, *poor remuneration and so you see people striking because of salaries*. *So who suffers*? *The pregnant mothers*, *are in labour and no one goes to the theatre for them*, *no one goes and attends the delivery*. ***#1 PNFP***

#### Health worker attitudes

Closely related to staff motivation, the poor attitudes of some of the health workers were observed as barriers to improved quality of MCH service delivery. Mandating health workers to implement evidence-based tools without paying attention to their concerns regarding a conducive work environment was reported to sometimes lead to inefficiencies in translating some of the evidence-based practices to respond to stillbirth risk factors;

*but the challenge is the attitude of health workers*. *You give them the knowledge but at the end of the day they still don’t change…*. *if the health workers do not believe in it they will not prescribe it for the mothers and yet it is very good but if you do not have that information you will not know*. ***#1 PFP***

#### Human resources governance

Within a decentralised health system, governance of human resources fell under the local government and public services while technical directives came from the Ministry of Health. The subnational priorities would sometimes not align well with the health sector aspirations especially as they related to addressing systems challenges. Under this arrangement, the accounting officer in the district is the Chief Administrative Officer (CAO) who is autonomous from the health sector. Variations in priorities would manifest from time to time as per context where recruitment of essential health workers is viewed in terms of the expenses incurred other than the critical role they add to the efficiency of the health systems responding to MCH risk factors including stillbirth;

*All the DHO’s are under the CAO and even the salary comes; so*, *when this DHO makes a budget and says*, *Mr*. *CAO*, *I want 2 anaesthetists*, *I want 4 midwives; that is doubling the wage*. *Where are you going to get the salary to pay them*? *And so*, *you have to work within what you have*. ***#2 Professional association***

#### Medical supplies

Several challenges underlie medical supplies and logistics. The bottlenecks within the medical supplies were mentioned as bottlenecks to the effective implementation of evidence-based interventions to address the stillbirth burden. As implementation progressed, attention to health systems input was manifesting. Aspects of inadequate medical products and supplies relative to demand were observed. In several of the cases, it was reflected as one of the factors impeding the optimal implementation of policies. Specifically, respondents called for a revision of the quarterly delivery of drugs which they observed as posing challenges impeding effective service delivery;

This National Medical Store business of delivering every quota and then when people have their money they give NMS when the quota is not finished; I think it is chaos. I think in that the districts should be empowered more. **#5 MOH**

The private-for-profit MCH service providers tended to lag in policy adoption relative to the public and PNFP. Within the private sector especially the lower level which offers maternity services, challenges of procuring drugs and other supplies without external funding/support were the lead cause. For example, the requirements to ensure coherent policy implementation presented a cost barrier to effective implementation occasioned by drugs and supplies expenses as reflected below;

*we are supposed to test for syphilis and syphilis is one of the commonest causes of stillbirths*. *It is a must but most of the [private] facilities don’t do it*, *why*? *Because they don’t have the reagents*, *they don’t have maybe the necessary laboratory supplies and things of that nature but it is on the antenatal card of women; it is a must*, *it is one of the investigations we must do*. ***#1 NGO***

Interruptions due to equipment breakdown were cited as other challenges. Respondents reported that some essential equipment such as oxygen cylinders and generators for the provision of power to supply the constructed theatres were sometimes dysfunctional. Other equipment was not repaired on time which occasioned service interruptions. In one of the scenarios, respondents reported a supply of mismatched oxygen cylinders and their regulators which disrupted service delivery. In another case, the generators to power the theatres would sometimes dysfunctional due to a lack of funds to buy fuel which led to referrals out for cases which would otherwise have been managed from within those health facilities;

*sometimes we would ask our health workers ‘why are you not referring to such a place*?*’*. *And they would say that they don’t have fuel for the generator that runs the theatre*, *they have to ask you to buy the fuel; those kinds of things and so some people would not want to go to nearer health facilities because of that and they go to the furthest*. ***#4 MOH***

#### Structural characteristics

Low levels of awareness about stillbirth risks by mothers were another barrier to the effective implementation of recommended evidence-based interventions. This was reported to affect in different ways including incidences of late reporting for both antenatal care and facility deliveries. Inconsistent or non-adherence to health promotion and stillbirth risk prevention behavioural advice by the mothers, as well as following guidance from health workers. This was not helped by the lean demand side component to address promotive behavioural change. Investments in the demand side component did not match the need for the same which affected the effectiveness of facility-level interventions. Respondents perceived a strong engagement in the community would have had an impact;

*But also*, *I think if the community program is strong I think it would produce a lot of impacts because it reaches individuals and not only women but all people in the community*, *and once people are convinced of what to do [they] can able to access the health facilities*. ***#5MOH***

#### Passive communities

Respondents revealed that the community empowerment component was slightly lagging compared to other facility-based interventions. Aspects reflective of inadequate empowerment of women to utilise available MCH services were reported. These were in areas of decision-making for timely care and financial vulnerability which caused the delay to seek care timely. Elements reflective of communities as passive recipients who could not exert accountability and oversight for better service delivery were still common.

*…*. *I would first focus on how can I empower the mother to seek health care and even detect those [danger] signs like early bleeding if I am pregnant and I have a drop of blood*. *She might take it lightly but it is a very big danger sign*. ***#2 Researcher***

The oversight functions, a key component of consistent policy implementation required more strengthening. common was the observation that with the introduced interventions, there were additional tasks and pressure to ensure every aspect of these interventions were implemented. Closer supervision at the regions to account for contextual variations was felt to be beneficial in responding to and improving the oversight functions aimed at addressing the stillbirth burden. Current arrangements for oversight aspects from the centre were perceived as a barrier and less effective.

*And people who are dealing with health*, *leaders for health should not have to travel long distances to the centre to get things done*. *The Ministry cannot go around this country so I think the Ministry has identified who their stakeholders are and whenever there is anything to be done they [should] call on the stakeholders*. ***#1Professional association***

#### Evaluation of interventions

Early evaluations to provide reality checks and feedback to implemented interventions are key. For this, stakeholders emphasised the need for timely feedback on whether the introduced policies were working as per plan. They felt that this would provide room within the health systems to absorb feedback to inform revisions hence facilitating early modifications.

*the policy is as good as its implementation so if we put up a policy and our best way of reviewing that is if we see the implementation changes because a policy needs to go with implementation then you can see if the policy has changed things*. ***#3MOH***

Due to inadequate funding, facility-based interventions were masking the importance of having a vibrant community arm. The inability for a substantial investment in the demand side was presented as a challenge to current programming towards addressing the stillbirth risk

*I think for us the interventions we do we want to concentrate mostly on the facility*. *We may not have a lot of community engagements although we encourage collaboration of course with the VHTs*. ***#1 Researcher***

Delivering interventions without due regard to regional contextual variations was observed as another challenge. There were regional variations in the prevalence of stillbirth and this was likely due to differences in implementation contexts. Whereas standardisation was a welcome health systems initiative, attention to context was also key to effective implementation. Respondents noted that other than solely relying on data for decision-making, input on contextual factors would be important in guiding the design of such intervention. Respondents attributed this to standard approaches used to interpret national data on the burden of stillbirth. They partly attributed it to the donor community which used this approach while designing interventions.

*I think the donor community has amicably contributed to the confusion in the healthcare system*. *The donor community looks at the computer; this is the methodology*, *these are process indicators*, *this is the what*, *and they don’t look at the actual place where things are going on*. ***#4MOH***

#### Infrastructure

The lower-level health facilities attended to a bigger bulk of mothers and children albeit with inadequate infrastructure. A common thread was that this posed a challenge of inconsistent policy implementation with low-quality service delivery among lower-level health facilities arising from adequate infrastructure;

*Infrastructure at lower facilities especially Health Center IIIs we still have a challenge because you find that they are trying to construct but most Center IIIs had no maternity although mothers go there to deliver and even when there are maternity services you may find………; but at least now they are improving*, ***#4Professional association***

### Processes

As technical aspects were strengthened, attention was being directed more to the quality and skills of the health workers and managers especially as it related to their ability to steer the subnational health systems into a functional one. Integration of proposed interventions within the available resources at the sub-national level was still a challenge in some areas. The planning and budgeting skills beyond the clinical/technical competencies were reported as requiring special attention. Many of the policy aspects required managerial competencies and decision-making. Respondents mentioned how this was reflected in managers’ ability to work within limited resources to ensure the interventions were implemented smoothly.

There were observed challenges to the effective coverage of available RMNCH interventions. Respondents noted that whereas some were supply-side systemic factors, others stemmed from the demand side. Despite the increased average uptake of antenatal care services, many users did not complete the recommended visits. There were still instances of mothers attending the recommended antenatal care visits but still would not deliver from the health facilities. Even for those that delivered from health facilities, negative outcomes were still being reported which meant that some risk factors were still sipping through the system.

*A lot of effort has been done to encourage mothers to come and deliver in health facilities but when they come they are losing their children*. *Antenatal coverage we are saying has gone up but when you look at antenatal 4 as to 1*, *the catchment is still below standard*. ***#2 MOH***

Some of the interventions, although implemented within the same health systems were not well aligned to provide a forward and backward linkage hence averting any possible stillbirth risks. This posed further challenges for the likelihood of having many interventions within the health systems that did not align well with each other and operated in parallel. A case in point was the nutrition interventions among adolescent girls and women.

Concerns emerged regarding interventions that focused on a single health worker. This was deemed to create work overload for the midwives within the maternity ward. This was feared to negatively affect the quality of maternal and child health services. It was observed that as health workers get overwhelmed policy implementation would be suboptimal due to fatigue.

*there is a lot of health worker fatigue because there are many programs that come in and they are focusing on the same health workers*. *So sometimes health workers have tended to place more emphasis on areas where maybe they have been motivated*; ***#1Professional association***

The continued implementation of pilots alongside mainstream policy translation of scaled-up interventions was observed as another likely barrier. Respondents noted that it was creating patches of “islands of success” within the same health systems which was producing different results. Different donors and implementing partners in some aspects continued to promote different priorities and interventions. In some frontline contexts, health workers would implement policy recommendations alongside project-supported pilots which came with varying incentives compared to standard routines on the sidelines.

*we also realized that they are overwhelmed by the interventions*, *everybody is doing something*. *So*, *I think it is also a problem that there are so many cooks in this kitchen that the food ends up not being okay*. ***#6 Professional association***

The inability to respond to other resource constraints at the frontline such as medical records was feared to create redundancies in the promoted policies. Respondents expressed concern that there was a likelihood for the guidelines distributed at the frontline to be shelved and not serve the intended purpose;

*For example*, *emphasis is again laid on this issue of the partograph but the use is not very okay*, *it is ill-used simply because some of these labour wards and hospitals get so many mothers and the number of health workers or midwives to carry out systematic WHO layout when you are conducting delivery…*..*they can’t follow it because of the number of the mothers*. ***#2 Professional association***

### Outputs

#### Quality-adjusted coverage

Whereas targeted interventions specifically responding to stillbirth was important, there was a need to reorient those towards the sexual and reproductive maternal newborn child and adolescent (SRMNCAH) health continuum of care towards the same. In addition, many of the risk factors which were outside the critical period of antenatal care, labour and delivery had not been adequately addressed which they felt had been adequately addressed such as nutrition. Even at implementation, it was observed that interventions that followed after birth would be prioritised even before the baby was born. This created incoherence in the implementation of interventions to address stillbirth risks as reflected in the quotation below;

*there is still work to do on strengthening or trying to address the causes of stillbirths because that is intricately related to how well the maternal health program is being run the reason for a mother to get a macerated stillbirth is something that has occurred in her antenatal period and so we are looking at what are the policies around malaria in pregnancy*, *that we need to work on*. ***#3 MOH***

#### Equity

An emerging challenge concerned equity in service provision. Less attention was paid to the referral system compared to facility-based interventions. Respondents noted that whereas the promoted interventions greatly relied on the tiered referral system for effective service delivery, it was not as heavily supported as the facility-based interventions. This was especially the case in regions/districts with less project support.

*So even if the doctor is available you cannot carry out the caesarean section*. *Then you go into the cycle of transferring the mother to the next level which is the district hospital and you may be far away from this health centre IV*, *transport is a problem; ambulances may be a problem and then she gets there and then there is a 3rd delay*. ***#1 Professional association***

#### Effectiveness

Rollout of evidence-based tools in isolation of streamlining other health systems inputs such as medicines and supplies, financing was observed as posing implementation challenges. Respondents noted that despite an emphasis on some of the evidence-based tools, they did not produce the expected output when the other health system’s inputs were not given priority.

*a partograph is used for monitoring when you are in labour*, *appropriate interventions are taken and of course*, *at the end of the day you can fall prey to all the building blocks*, *the health care system*, *financing and all those things but I think we need to streamline interventions*. ***#6 Professional association***

The quality of MCH services was increasingly becoming a concern because many of the deaths were occurring within the health facilities. It was observed that where many of the interventions were complementary, the rates of reduction were varying between child and neonatal mortality. Respondents contend that this was likely to affect the effectiveness of some interventions.

*and we have noticed that although the child mortality rate has gone down*, *the newborn mortality has not gone down as fast and we think that it is an area that needs a lot of our attention and focus*. ***#5 Professional association***

The risk of intervention relapse emerged as a potential challenge that was likely to derail the successes so far achieved. Respondents observed that at the time, the country’s momentum to address the stillbirth burden was visible in most aspects.

*I pray we don’t lose the momentum as a country and continue progressing and the next DHIS I am sure we shall see much more because there was already a drop in maternal mortality*. ***#2 Researcher***

## Discussion

The study explored the national-level key stakeholders’ perspectives regarding intervention progression and emerging challenges towards the national stillbirth reduction strategic response within a Ugandan context. Overall, they perceived the interventions as timely and with the potential to lead to the desired outcomes in addressing the stillbirth burden. Emerging challenges arising from the implementation strategies were observed. The approach in responding to these challenges was likely to impede their effectiveness. In the section that follows, we attempt to interpret and discuss the implications of the key findings.

### Inputs

Health systems inputs are key to the successful implementation of interventions responding to a public health issue. The absence of essential inputs will impact the level of the health system’s effectiveness [26]. Besides, they demonstrate the level of the health system’s readiness to implement policy recommendations [27]. Our results revealed that although efforts were made to prepare the health systems with many essential inputs to implement evidence-based interventions, some challenges regarding the interventions were persisting.

One of the key findings revealed was the slow recruitment of the required human resource to implement evidence-based interventions to address stillbirth. This observation may have been due to the mismatch or uncoordinated planning within the different government ministries responsible for resource allocation, recruitment and deployment of health workers. Whereas the Ministry of Health (MoH) may have played its part to highlight and address the human resource challenges for preparation of the health systems to implement interventions to respond to the stillbirth burden, recruitment and allocation of finances to facilitate recruitment and subsequent remuneration of human resources was vested outside the ministry of health mandate. Whereas recruitment falls within the health service commission mandate, appointment into public service is by the Ministry of Public Service and their remuneration pretty much rests with the Ministry of Finance Planning and Economic Development mandate. The country has witnessed on-and-off freezes on staff appointments into public service with the current health service staffing structure a mandate under MoH said to be limited about the service delivery mandates. This has coincided with the shrinking budgetary allocation for health from the government which continues to reduce concerning the Abuja declaration recommendation of at least 15% of the national budget. This points to the need for well-coordinated planning and management of intersectoral collaboration if such interventions are to succeed when translated into routine services [28]. It also highlights an important revelation that addressing the stillbirth burden thrives well in a multisectoral approach just like any other maternal and child health intervention [29]. Elsewhere, the value of a multisectoral approach towards addressing public health challenges has been emphasised [28]. within maternal and child health programming, it has been highlighted as an essential ingredient in responding to service delivery bottlenecks [30].

Another key finding was the health workers’ motivation and attitudes. It emerged that both motivating the health workers and their attitudes towards implementation of the promoted interventions were in some ways a challenge. It impacted the effective delivery of evidence-based interventions to address stillbirth. Overall, the factors which affected health worker motivation were the low wages relative to the workload expectations and the inadequate staff accommodation onsite which affected their full-time delivery of maternal and child health services including interventions responding to stillbirth risks. This may have been the case because whereas there are efforts to improve service delivery to address stillbirth through the implementation of evidence-based interventions, resource allocation and focus were not evenly dedicated between policy compliance and other workplace environmental bottlenecks which were likely to affect staff motivation such as accommodation and commensurate salary enhancements. Intrinsic motivation of health workers is a key factor for the effective delivery of health services when considering the realisation of policy objectives [31]. More so, it is even paramount when previously piloted interventions get rolled out within routine health services [32]. Within the Ugandan context, a study conducted by Källander et al shared similar findings where the value of intrinsic motivation of health workers through supportive supervision meetings was highlighted as important for their productivity [33]. Factors like staff accommodation, recognition and appreciation of tasks performed, being availed with adequate resources to provide the required care and fair remuneration were particularly singled out as important for better health service delivery [34]. Indeed, for years now, the MoH has prioritised addressing staff accommodation in efforts to improve health service delivery [35–37]. It is therefore important that while focusing on the rollout of evidence-based interventions to respond to the stillbirth burden it was equally important to pay equal attention to other health systems factors which were likely to affect the productivity of health workers such as salaries and accommodation [35, 36].

Another key finding from our study was that inadequacies in medical supplies impeded the realisation of the policy goals. Respondents mentioned that there were instances when they would be faced with stockouts of supplies which impeded policy implementation. The UN commission designated a basket of essential RMNCH commodities which need to be prioritised to facilitate the implementation of evidence-based interventions [38]. Indeed, the MoH went ahead to localise these recommendations into the routine standard of care where the specific commodities were included in the list of essential medicines to be supplied [39]. Perhaps the observed mismatch could have been brought about by the logistical bottlenecks to the last-mile delivery of drugs and medical supplies in addition to ordering and procurement bureaucracies within the existing practices [26]. This has for some time persisted as a major challenge causing drug stockouts at the final points of health service delivery [40]. From this observation, the key lesson revealed is that for interventions to be effective, there is a need to pay attention to both the inputs such as medicines as well as the promoted guidelines. The requirement to strike a balance is key. The volumes and availability of drugs and medical supplies are essential for the effective implementation of policy recommendations.

### Processes

Health managers often faced bottlenecks in integrating new interventions within routine care for the effective delivery of quality healthcare. The promoted evidence-based interventions not only required guideline adherence not only from the service providers but also from the service users. The inability to complete the recommended antenatal care visits and delivery within the health facilities compromised the effectiveness of the promoted interventions. This could have been due to variations in resource availability and allocation to support their implementation between effective pilots and when they were integrated into routine health services. It is especially the case since small pilots tend to be heavily resourced and post impressive results while routine services suffer from chronic resource constraints to support their implementation [32]. Elsewhere, many programs which were found to be effective during pilots tended to fail when taken to scale [41]. This poses a challenge hence leading health systems into a vicious cycle of “pilotitis” (an expression of frustration due to the inability to scale up successful pilots) in the quest to prove that certain interventions work [42, 43]. It remains a major challenge to the sustainability of many interventions which start as pilots in low- and middle-income countries. The implementation context is one attribute that tends to lead to such results [41] as they vary from pilots to real-life implementation. Integration of interventions into routine care requires careful attention to facilitate a seamless transition process. Service users’ feedback and cooperation for the effective implementation of the promoted interventions are as well important. While designing interventions, focusing on the demand side to incorporate user feedback and interests would address some of the barriers while securing their commitment to collaborate on the promoted interventions [40].

### Outputs

Despite the translation of interventions into routine care, incidences of stillbirth continued to occur within the health facility settings. It negatively impacted the expected outcomes of these interventions and policy provisions. This was likely due to causes and risk factors which rested outside the critical periods of antenatal care, labour and delivery. Broadly these speak to the social determinants of health. This may have been the case because most of the prioritised interventions save for data and registration systems focussed on the critical periods when most risks are heightened. Due to resource constraints, minimal attention was paid to responding to risk factors that were outside the critical periods such as risk factors along the reproductive maternal life course. This may be the case because of the complexity and the many intervening factors contributing to the eventual risks that would need to be attended to when such interventions are prioritised. Besides, such an intervention would require more investment targeted and “whole of health” and yet results would only be visible in the long term after consistent implementation processes [44, 45]. Such interventions are difficult to secure funding commitment for the duration when results would be realised. Given the current donor and the funding terrain, there tends to be a preference for programs that can demonstrate feasibility within the short and medium term. The complexity of social behavioural interventions has been emphasised before [46, 47]. Whereas social behavioural interventions do not easily secure short-term gains, their benefits in addressing public health challenges in the long term ought not to be compromised. It is therefore imperative that governments and donors equally give priority to the social determinants of health when responding to the stillbirth burden in the long term.

### Limitation

This study is not without limitations; first, the purposive sampling and the limited duration of the study may have left out perspectives of other key actors, and therefore the results reported here may have represented a one-sided story more so from health workers directly involved in the implementation of MCH. Secondly, the study relied on information provided by the respondents, and in the context where a lot goes on in the implementation of maternal and child health programs, views may be subjected to a recall bias. Therefore, in the absence of documented experiences, we were unable to triangulate the respondents’ information. Third, there was a risk of social desirability associated with research related to program implementation experiences where respondents may systematically edit out views that may appear to reflect the poor performance of entities they represent. A key strength of this study is that the analysis and discussion draw on perspectives from multiple sources of data including both national-level respondents and key strategic documents and reports related to the implementation of maternal and child health policy in the country. This enabled the triangulation of information provided from more than one source which ensured the reliability of the information collected. Besides, the analysis was conducted deductively drawing on the main study themes, where particular text fitting into a specific theme was applied.

#### Policy implications

Early evaluations of these policy aspects would go a long way to identify the performance of particular components to inform revisions and modifications.

#### Implications for practice

Attention to context particularly the limited resource setting is crucial beyond the emphasis on outcomes from pilots. The emerging lessons from the health systems’ response to the introduction of these interventions could be a lesson to inform future programs and approaches to MCH service delivery improvements.

#### Implications for research

From the lessons we have learnt in this study, we would recommend another follow up study that evaluates each of the intervention components and how the health system responded to its introduction in different contexts.

## Conclusion

In this exploratory study, national-level stakeholders perceive the adopted stillbirth reduction strategies as having the potential to address the burden. They however highlight potential challenges which ought to be addressed and opportunities to explore potential solutions befitting the national-level context. Continuous learning and adjusting of intervention implementation will be key in unlocking the potential of these evidence-based interventions and tools towards addressing the national stillbirth burden. While solutions to the current health systems challenges are awaited, our conclusion advocates for a more dedicated focus to improve learning at the frontline where the interface with mothers occurs.

## List of abbreviation

ANC: Antenatal Care
DHIS: District Health Information System
CAO: Chief Administrative Officer
CEmONIC: Comprehensive Emergency Obstetric and Neonatal Intensive Care
COREQ: Consolidated Criteria for Reporting Qualitative Research
CQI: Continuous Quality Improvement
IPMR: Institutional Perinatal Mortality Rate
LMIC: Low- and Middle-Income Countries
MPDSR: Maternal and Perinatal Death Surveillance and Responses
NDP: National Development Plan
NGOs: Non-Governmental Organisations
RMNCAH: Reproductive Maternal Child and Adolescent Health
UHC: Universal Health Coverage
WHO: World Health Organisation
